# Chronic intermittent hypoxia increases rat sternohyoid muscle NADPH oxidase expression with attendant modest oxidative stress

**DOI:** 10.3389/fphys.2015.00015

**Published:** 2015-01-30

**Authors:** Robert Williams, Paul Lemaire, Philip Lewis, Fiona B. McDonald, Eric Lucking, Sean Hogan, David Sheehan, Vincent Healy, Ken D. O'Halloran

**Affiliations:** ^1^Department of Physiology, School of Medicine, University College CorkCork, Ireland; ^2^School of Medicine and Medical Science, University College DublinDublin, Ireland; ^3^School of Biochemistry and Cell Biology, University College CorkCork, Ireland

**Keywords:** apocynin, intermittent hypoxia, NADPH oxidase, oxidative stress, respiratory muscle, sternohyoid, sleep apnea, upper airway

## Abstract

Chronic intermittent hypoxia (CIH) causes upper airway muscle dysfunction. We hypothesized that the superoxide generating NADPH oxidase (NOX) is upregulated in CIH-exposed muscle causing oxidative stress. Adult male Wistar rats were exposed to intermittent hypoxia (5% O_2_ at the nadir for 90 s followed by 210 s of normoxia), for 8 h per day for 14 days. The effect of CIH exposure on the expression of NOX subunits, total myosin and 4-hydroxynonenal (4-HNE) protein adducts in sternohyoid muscle was determined by western blotting and densitometry. Sternohyoid protein free thiol and carbonyl group contents were determined by 1D electrophoresis using specific fluorophore probes. Aconitase and glutathione reductase activities were measured as indices of oxidative stress. HIF-1α content and key oxidative and glycolytic enzyme activities were determined. Contractile properties of sternohyoid muscle were determined *ex vivo* in the absence and presence of apocynin (putative NOX inhibitor). We observed an increase in NOX 2 and p47 phox expression in CIH-exposed sternohyoid muscle with decreased aconitase and glutathione reductase activities. There was no evidence, however, of increased lipid peroxidation or protein oxidation in CIH-exposed muscle. CIH exposure did not affect sternohyoid HIF-1α content or aldolase, lactate dehydrogenase, or glyceraldehyde-3-phosphate dehydrogenase activities. Citrate synthase activity was also unaffected by CIH exposure. Apocynin significantly increased sternohyoid force and power. We conclude that CIH exposure upregulates NOX expression in rat sternohyoid muscle with concomitant modest oxidative stress but it does not result in a HIF-1α-dependent increase in glycolytic enzyme activity. Constitutive NOX activity decreases sternohyoid force and power. Our results implicate NOX-dependent reactive oxygen species in CIH-induced upper airway muscle dysfunction which likely relates to redox modulation of key regulatory proteins in excitation-contraction coupling.

## Introduction

Obstructive sleep apnea syndrome (OSAS) is a debilitating oxidative stress disorder (Lavie, 2003) which is very common. OSAS is associated with a wide spectrum of co-morbidities including cardiovascular, metabolic and neurocognitive dysfunction (Verstraeten, 2007; Lévy et al., 2009). Chronic intermittent hypoxia (CIH) is a central dominant feature of OSAS, and there is now convincing evidence from animal models that CIH recapitulates many of the hallmark features of the disorder. Upper airway (UA) muscle dysfunction is implicated in the pathophysiology of OSAS. OSAS patients (Carrera et al., 1999) and the English bulldog (Petrof et al., 1994), an animal model of OSAS, show signs of UA dilator muscle remodeling, dysfunction and damage. CIH alters UA muscle function (McGuire et al., 2002; Liu et al., 2005; Pae et al., 2005; Dunleavy et al., 2008; Ding and Liu, 2011; Skelly et al., 2012a) and induces UA muscle structural remodeling in some (McGuire et al., 2002; Pae et al., 2005) but not all (Ray et al., 2007; Skelly et al., 2012a) studies.

CIH exposure is typically associated with an increased production of reactive oxygen species (ROS) (Prabhakar, 2001; Peng and Prabhakar, 2003; Yuan et al., 2004; Shan et al., 2007; Dunleavy et al., 2008; Dutta et al., 2008; Raghuraman et al., 2009; Sharma et al., 2009; Khan et al., 2011) which can contribute to the development of skeletal muscle dysfunction (Dunleavy et al., 2008; Dutta et al., 2008; Jackson, 2008; Ding and Liu, 2011; Skelly et al., 2012a; Shortt et al., 2014). Pro-oxidants exacerbate (Dunleavy et al., 2008), while antioxidant strategies ameliorate (Dunleavy et al., 2008; Skelly et al., 2012a; Shortt et al., 2014) CIH-induced respiratory muscle dysfunction. Moreover, superoxide scavengers increase sternohyoid muscle force (Skelly et al., 2010, 2012b) highlighting that basal ROS production is inhibitory to UA muscle function. These observations highlight that ROS are important modulators of respiratory muscle performance under physiological and pathophysiological conditions. Although it is well established that CIH increases ROS production, the source of ROS is less clear. CIH-induced oxidative stress in the liver (Jun et al., 2008), cardiovascular system (Nisbet et al., 2009), and brain (Hui-guo et al., 2010) is ameliorated by the inhibition of NADPH oxidase (NOX) activity suggesting that this membrane-bound superoxide generating enzyme is a major source of CIH-induced ROS. In skeletal muscle fibers, NOXs are localized to the sarcoplasmic reticulum (Xia et al., 2003), transverse tubules (Hidalgo et al., 2006), and plasma membranes (Javesghani et al., 2002); inhibition of NOXs in skeletal muscle reduces the levels of superoxide (Javesghani et al., 2002; Patwell et al., 2004). We recently reported that CIH-induced diaphragm dysfunction in the rat is blocked by chronic supplementation with the putative NOX inhibitor—apocynin (Shortt et al., 2014). Together, these studies implicate a role for NOX-derived ROS in skeletal muscle (dys) function.

We sought to determine the effects of CIH on the expression levels of NOX proteins in rat sternohyoid muscle (a representative UA dilator critical in the control of airway caliber) and to establish whether or not CIH alters the redox status of the sternohyoid. In functional studies of sternohyoid muscle *ex vivo*, we explored the effects of apocynin on isometric and isotonic contractile properties. We postulated that CIH upregulates NOX expression in rat sternohyoid muscle resulting in oxidative stress and that NOX-dependent ROS exert an inhibitory effect on sternohyoid force- and power-generating capacity.

## Materials and methods

### Ethical approval

All procedures involving animals were performed under license from the Irish Government Department of Health and Children and were carried out in accordance with National and European guidelines, following approval from University College Dublin Animal Research Ethics Committee.

### Chronic intermittent hypoxia

Adult male Wistar rats were exposed to sham or chronic intermittent hypoxia (CIH) exposure. Rats were housed as normal in standard cages placed within commercially designed environmental chambers (Oxycyler™, Biospherix, USA) for daily gas treatments. Ambient oxygen was servo-controlled to generate intermittent hypoxia: 90 s hypoxia (5% O_2_ at the nadir) and 210 s normoxia (21% O_2_), 12 cycles per hour, 8 h per day for 14 consecutive days. A rodent pulse oximeter (Mouse Ox™ Starr Life Sciences Corp., USA) was used to determine the resulting arterial oxygen saturation (SaO_2_) during CIH exposure; SaO_2_ decreased to ~70–80% at the nadir of the recurrent hypoxic events. On the day following gas treatments, animals were euthanized humanely (cervical spinal transection under 5% isoflurane anesthesia) and the sternohyoid muscles were excised.

### Tissue preparation

Muscles were homogenized (GLH Homogeniser Omni International) in RIPA lysis buffer (25 mM Tris-HCl pH 7.6, 150 mM sodium chloride, 1% NP-40, 1% sodium deoxycholate, 0.1% SDS) supplemented with protease inhibitor cocktail (1 mM AEBSF, 800 μ M aprotinin, 40 μ M bestatin, 14 μ M E-64, 20 μ M leupeptin, 15 μ M pepstatin A; Sigma Aldrich, Ireland) and phosphatase inhibitors 10 mM sodium fluoride, 1 mM sodium orthovanadate, using a 10% w/v ratio. Samples were homogenized using 8 × 10 s bursts and left on ice for 20 min. Homogenates were then centrifuged for 15 min at 11,000 g and the supernatants were stored at −80°C. Protein concentration was determined using a bicinchoninic (BCA) assay (Thermo Scientific, Ireland) as described in the manufacturer's protocol with absorbances measured at 562 nm using a SpectraMax-M3 spectrophotometer (Molecular Devices, USA).

### Western blotting

Protein samples were mixed with an equal volume of 2x laemmli buffer (4% sodium dodecyl sulfate (SDS), 10% β-mercaptoethanol, 20% glycerol, 0.004% bromophenol blue, 0.125 M Tris-HCl, pH 6.8) and 15 μ g of protein from each sample was resolved on 7.5 and 12.5% SDS-polyacrylamide gels depending on the protein. Resolved proteins were then electrophoretically transferred onto nitrocellulose membranes (Bio-Rad, Hercules, CA). Membranes were incubated in 0.1% (w/v) Ponceau S in 5% acetic acid to reversibly stain the transferred proteins to assess equal protein loading and transfer, and were digitally photographed for densitometric analysis. The membranes were blocked for 1 h in TBST (20 mM Tris-HCl, pH 7.6, 150 mM NaCl, 0.1% Tween) containing 5% non-fat dried milk and were incubated overnight with the primary antibody specific for the protein of interest as follows: anti-NOX 2 and anti-p47 phox, 1:1000 (BD Biosciences, UK); anti-p22 phox, 1:1000 (Santa Cruz, USA); anti-4-HNE, 1:2000 (Millipore, Ireland).

Membranes were incubated for 1 h at room temperature with a 1:2000 dilution of HRP-linked anti-rabbit or anti-mouse secondary antibody (Cell Signaling Technology, USA) in 5% non-fat dried milk/TBST depending on the primary antibody of interest. Bands were visualized using enhanced chemiluminescence (ECL Plus, GE Healthcare, UK) and exposure to chemiluminescent sensitive film (Kodak, USA). Films were developed, digitally photographed and densitometric analysis of bands of interest was performed (QuantityOne, Biorad). Band intensities of proteins of interest were normalized to the intensities of the corresponding Ponceau S staining proteins which were also measured by densitometric analysis to adjust for protein loading, allowing comparative analysis between sham and CIH-exposed muscles.

### Protein free thiol and carbonyl group content

Muscle homogenates were incubated with either 2 mM iodoacetamidofluorescein (IAF) or 2 mM fluorescein-thiosemicarbazide (FTSC) (Sigma-Aldrich Co., Ireland) for 2 h in the dark on ice for detection of protein free thiol and carbonyl groups, respectively. Samples were then precipitated with 20% trichloroacetic acid (TCA) in acetone, followed by centrifugation at 11,000 g at 4°C for 3 min. Protein pellets were then washed with ice-cold excess 1:1 ethylacetate/ethanol or acetone (for FTSC and IAF respectively) to remove excess TCA, interfering salts and non-protein contaminants. Samples were dried, re-suspended in sample buffer containing 5% β-mercaptoethanol and heated at 95°C for 5 min before 1D electrophoretic separation on a 12% polyacrylamide gel. Fluorescent images of the gels were captured on a Typhoon Trio+ Variable-Mode Imager (GE Healthcare, UK). Protein bands were visualized by colloidal coomassie staining and images were captured on a calibrating image densitometer (GS-800, Bio-Rad, USA).

### HIF-1α and metabolic enzyme activities

Sternohyoid HIF-1α content was assayed by an immuno-linked luminescence assay in accordance with manufacturer's instructions (Mesoscale Discovery, Gaithersburg, USA). HeLa cells treated with and without cobalt chloride for 16 h provided positive and negative controls respectively for the HIF-1α assay. Aconitase activity was measured using a colorimetric reaction in accordance with the manufacturer's instructions (Abcam, Cambridge, UK). Aconitase is a TCA cycle enzyme that catalyzes the isomerisation of citrate to isocitrate. Isocitrate, in this assay, undergoes further biochemical reaction resulting in a product that converts a nearly colorless probe into an intensely colored form with a peak absorbance at 450 nm. Samples were incubated in an activating solution containing cysteine-HCl and (NH_4_)_2_Fe(SO_4_)_2_on ice for 1 h before addition to a 96-well plate in duplicate along with isocitrate standards. One half of the duplicate wells received the sample reaction mixture (containing assay buffer, enzyme mix and substrate) and the other half the background mixture (containing assay buffer and enzyme mix only) and samples were incubated for 60 min at 25°C. Developer was then added to each well and samples were incubated for a further 10 min. Absorbance was measured at 450 nm and background was subtracted from the test sample. One unit of aconitase activity is the amount of enzyme that will isomerize 1 mmoles of isocitrate per minute at pH 7.4 and 25°C. Fructose-1, 6-bisphosphate Aldolase A (aldolase) is the isoform of the enzyme found predominantly in skeletal muscle. It is the 4th enzyme of the glycolysis pathway which catalyzes the conversion of fructose-1-6-bisphosphate into both 3-phosphoglyceraldehyde and dihydroxyacetone phosphate. We developed an assay based upon Boyer's modification of the hydrazine assay where 3-phosphoglyceraldehyde reacts with hydrazine to form a hydrazone which absorbs at 240 nm. 4 mM fructose-1,6-bisphosphate (pH 7.5) (25% v/v), 0.03 mM EDTA pH 7.5 (25% v/v), and 2.3 mM hydrazine sulfate (25% v/v) were added to a 96-well plate and absorbance was recorded for 10 min. Samples and a dH_2_O blank (25% v/v) were then added to the plate and absorbance was recorded for another 10 min. Using the linear portions of the curve, the A_240_/min of the blank was subtracted from the A_240_/min of the test. One unit is described as a change in absorbance of 1.00 per minute at 25°C and pH 7.5. For GAPDH activity measurement, samples were added to 13.5 mM sodium pyrophosphate buffer (pH 8.5) containing 30 mM sodium arsenate, 0.25 mM NAD with 3.325 mM DTT. Samples were incubated at 25°C for 10 min to achieve temperature equilibration and to establish a blank rate, if any. 0.5 mM DL-glyceraldehdye-3-phosphate was added and absorbance was recorded for 10 min at 339 nm. Measured rates were corrected by measuring the blank rate of the reaction. One unit is defined as 1 μmol NADH generated per minute. Citrate synthase activity was determined using a commercial kit (Sigma) as per manufacturer's instructions. Citrate synthase catalyzes the reaction between acetyl coenzyme A (acetyl CoA) and oxaloacetic acid (OAA) to form citric acid and CoA with a thiol group which reacts with DTNB in the reaction mixture to form 5-thio-nitrobemzoic acid (TNB) which is absorbed at 412 nm. One unit causes the synthesis of one micromole of citrate per minute per mg protein at 25°C and pH 7.5.

### Muscle physiology

Adult male Wistar rats were anesthetized with 5% isoflurane by inhalation in oxygen and killed by cervical spinal transection. Sternohyoid muscles were excised and longitudinal bundles were suspended vertically with fine (non-elastic) string; one end of each strip was mounted to tissue holders, while the other end was tied firmly to a hook, which sat on a dual-mode force transducer allowing assessment of isometric and isotonic properties. The muscle fiber bundles affixed to the tissue holders were then suspended in standard water-jacketed tissue baths. The tissue baths were filled with Krebs salt solution, maintained at 35°C and bubbled with 95% O_2_ and 5% CO_2_. The Krebs solution contained: 120 mM NaCl, 25 mM NaHCO_3_, 12 mM MgSO_4_, 1.2 mM NaH_2_PO4, 2.5 mM CaGluconate, 5 mM KCl, and 11.5 mM Glucose. D-tubocurarine (25 μM) was used in all experiments to exclude any potential involvement of excitation of intramuscular nerve branches. The muscles were stimulated using supramaximal square wave constant current stimulators delivered via two platinum electrodes which flanked the tissue in the bath. The change in tension was transduced, amplified and converted from an analog-to-digital signal where it was displayed and recorded on a computer for later analysis. Optimum muscle length (L_o_) was determined by repeated twitch stimulation while adjusting the length of the muscle with the micropositioner. The muscle preparations remained at L_o_ for the duration of the study. Studies were conducted in the absence and presence of the putative NOX inhibitor, apocynin (2 mM). Muscle preparations were allowed to equilibrate for 10 min before starting the experimental protocol.

#### Protocol

Isometric twitch force and kinetics (contraction time and half-relaxation time) were determined with the lever arm of the force transducer set to maximum rigidity (~500 mN; >100% load). Next, an isometric tetanic contraction (F*max*) was elicited by stimulating at 100 Hz for 300 ms (O'Halloran, 2006; Skelly et al., 2010, 2012a,b; Shortt et al., 2014). Following a 5 min rest period, concentric contractions were elicited in incremental steps with varying load (0.1, 1, 5, 10, 15, 20%; % of F*max*) with 1 min rest between each contraction. Muscle length returned to L_o_ following each contraction. Shortening (S*max*) was determined as the maximum distance shortened during contraction. Peak shortening velocity (V*max*) was determined as distance shortened during the initial 30 ms of shortening, when velocity is greatest at 0% load. Mechanical power was determined at each step of the incremental load step test as the product of force x shortening velocity.

### Data analysis

Peak specific force (F*max*) was calculated in N/cm^2^ of muscle cross-sectional area. Cross-sectional area was calculated as follows: mass (g)/(Lo (cm) × muscle density (g/cm^3^); muscle density was estimated to be 1.056 g/cm^3^. Maximum shortening (S*max*) was calculated as length shortened per optimal length (L/L_o_). Peak shortening velocity (V*max*) was calculated as L_o_/s. Peak mechanical power (P*max*) was calculated as Watts/cm^2^.

### Statistical analysis

Data are expressed as mean ± SEM. Statistical comparisons were performed between groups using Student unpaired *t*-tests, Mann Whitney *U*-tests, or Two-Way ANOVA with Bonferroni *post-hoc* tests as appropriate using Graph Pad Prism. *P* < 0.05 was the criterion for statistical significance in all tests.

## Results

### Effect of CIH on sternohyoid NOX subunit and myosin expression

**Figure 1:**
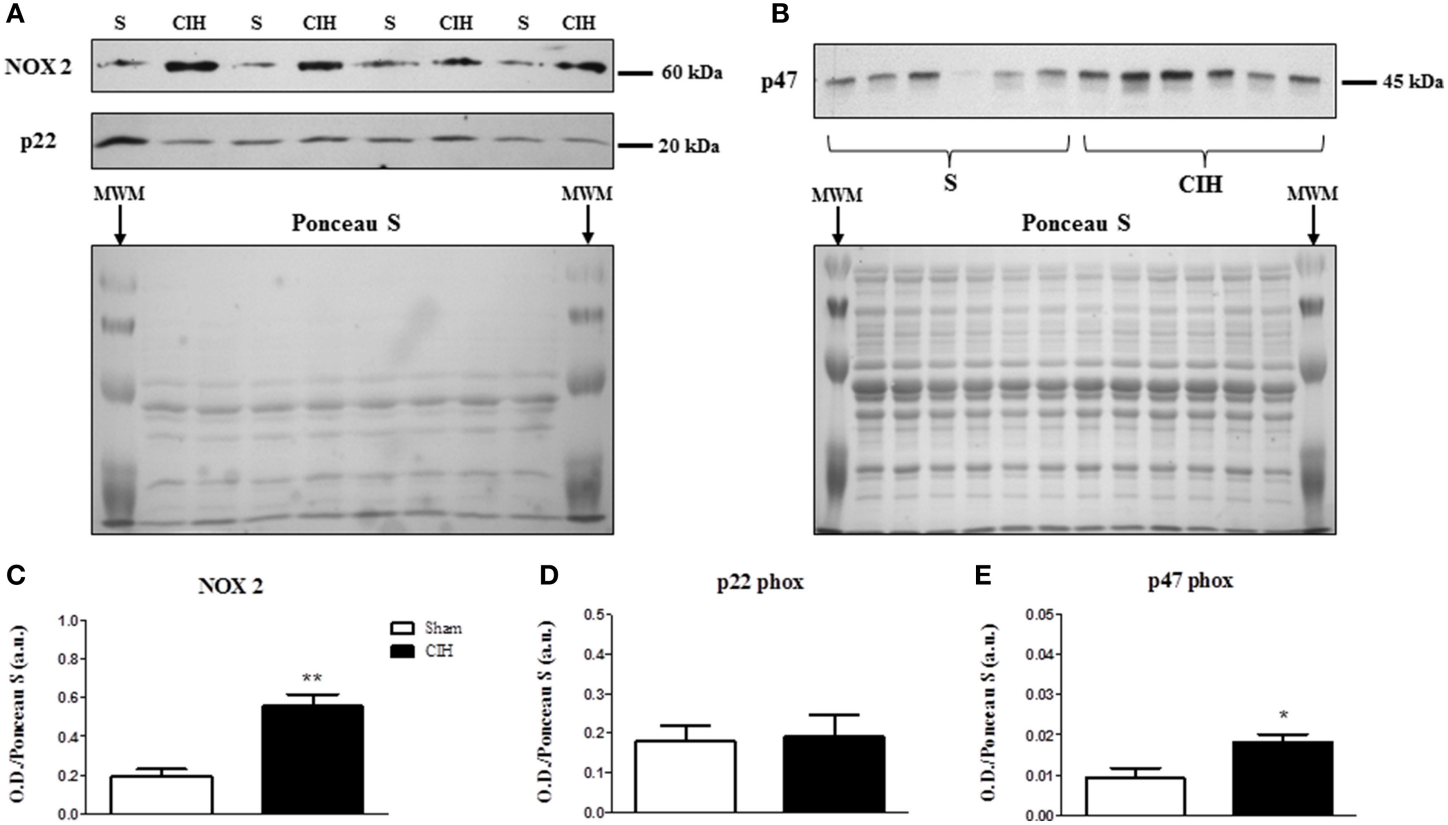
**Western blot of NOX subunit expression in the sternohyoid muscle from sham (S) and chronic intermittent hypoxia (CIH)-exposed rats**. **(A)** Expression of the catalytic superoxide-generating NOX 2 subunit and the co-anchoring p22 phox subunit with the corresponding Ponceau S stained membrane used to normalize protein loading and electro-transfer. MWM = pre-stained molecular weight markers. **(B)** Expression of the organizer subunit, p47 phox with corresponding Ponceau S stained membrane. Densitometric analysis of NOX subunit band intensities normalized by densitometric intensities of corresponding Ponceau-S stained proteins expressed in arbitrary units (a.u.) are shown in **(C–E)**. **(C)** A near 3-fold increase in NOX 2 expression was observed in the CIH-exposed group compared to sham control (^**^P = 0.002; Student unpaired *t*-test, *n* = 4 per group). **(D)** No change in the p22 phox subunit expression was observed (*P* = 0.884, *n* = 4 per group). **(E)** A near 2-fold increase in the p47 phox subunit expression was observed in the CIH-exposed group compared to sham control (^*^P = 0.014, *n* = 6 per group). Values are mean ± SEM.

We postulated that CIH would increase sternohyoid NOX subunit expression. Western blots are shown in Figures 1A,B. CIH significantly increased NOX 2 (Figures 1A,C) and p47 phox (Figures 1B,E)—but not p22 phox (Figures 1A,D)—expression in rat sternohyoid muscle. Total myosin protein content was unaffected by CIH exposure (not shown).

### Effect of CIH on sternohyoid 4-HNE protein adduct levels and protein free thiol and carbonyl group content

**Figure 2:**
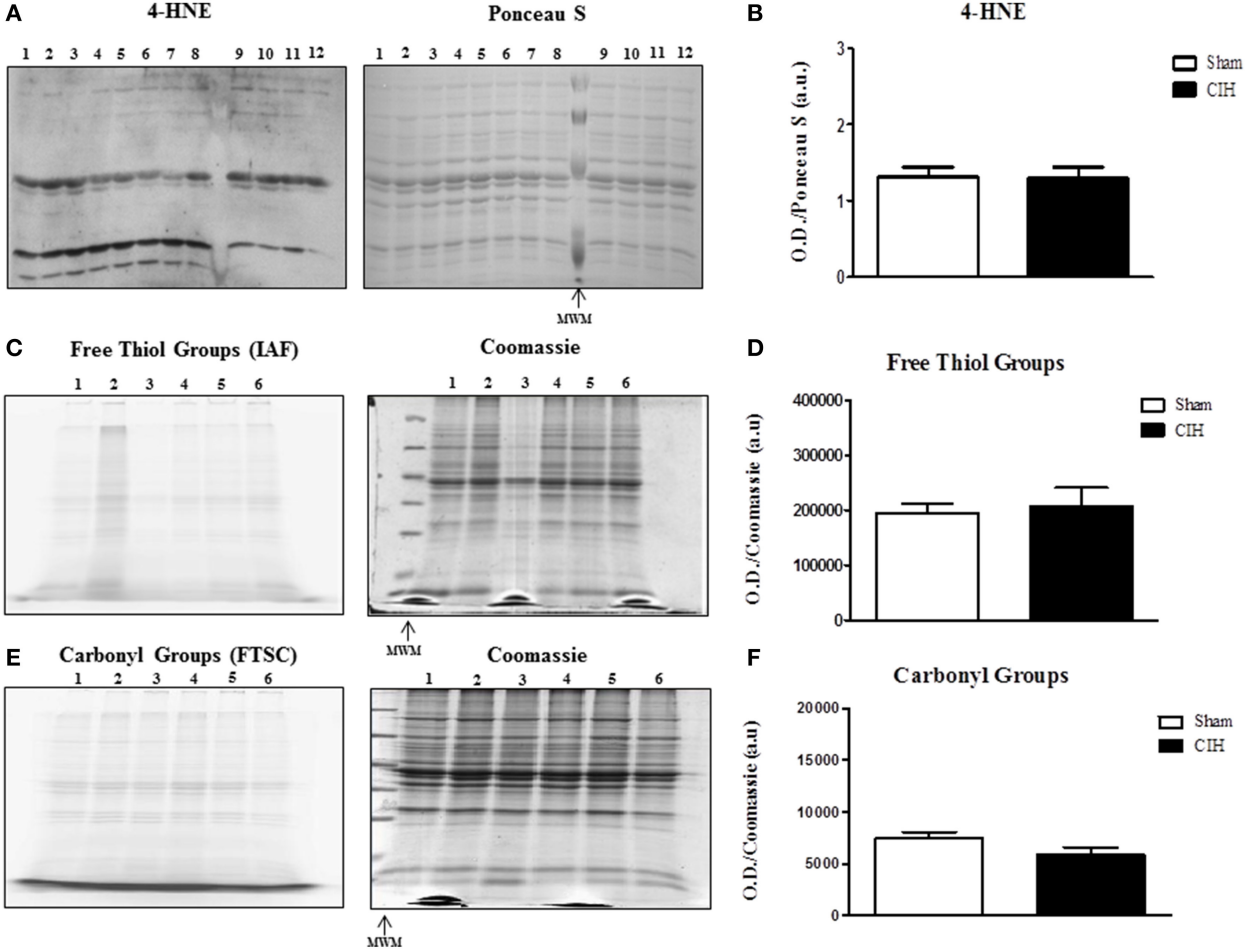
**Western blot of 4-HNE protein adducts and 1D electrophoresis to determine free thiol and carbonyl group content in the sternohyoid muscle from sham and chronic intermittent hypoxia (CIH)-exposed rats**. Sham and CIH samples were loaded in alternate lanes (numbered in the figure). **(A)** Sternohyoid 4-HNE content together with Ponceau S stained membrane. **(B)** Densitometric analysis of 4-HNE protein adduct band intensities normalized by densitometric intensities of corresponding Ponceau S stained proteins expressed in arbitrary units (a.u.) from sham and CIH-exposed rats. No significant difference was observed in 4-HNE protein adduct content (*P* = 0.9372; Student unpaired *t*-test) comparing sham and CIH-exposed sternohyoid muscles. Representative images of sternohyoid iodoacetamidofluorescein (IAF)-tagged protein free thiol groups **(C)** and fluorescein-thiosemicarbazide (FTSC)-tagged carbonyl groups **(E)** with corresponding coomassie stained membranes used to normalize protein loading and electrotransfer. Densitometric analysis of protein free thiol group **(D)** and carbonyl group **(F)** contents normalized by densitometric intensities of corresponding coomassie stained proteins expressed in arbitrary units (a.u.) from sham and CIH-exposed rats. Protein free thiol and carbonyl group content of the sternohyoid muscle was not significantly different in sham and CIH-exposed rats (*P* = 0.699 and *P* = 0.180 respectively; Student unpaired *t*-tests). Values are mean ± SEM; *n* = 6 per group.

We postulated that CIH would increase lipid peroxidation and protein oxidation. Western blot with respective Ponceau S stained gel is shown in Figure 2A. CIH exposure did not affect sternohyoid 4-HNE protein adduct levels (Figure 2B). Representative fluorescent 1D gels tagging protein free thiols (Figure 2C) and protein carbonyls (Figure 2E) are shown with respective coomassie stained gels. CIH exposure did not affect protein free thiol (Figure 2D) or carbonyl group (Figure 2F) content.

### Effect of CIH on sternohyoid muscle aconitase and glutathione reductase activities

**Figure 3:**
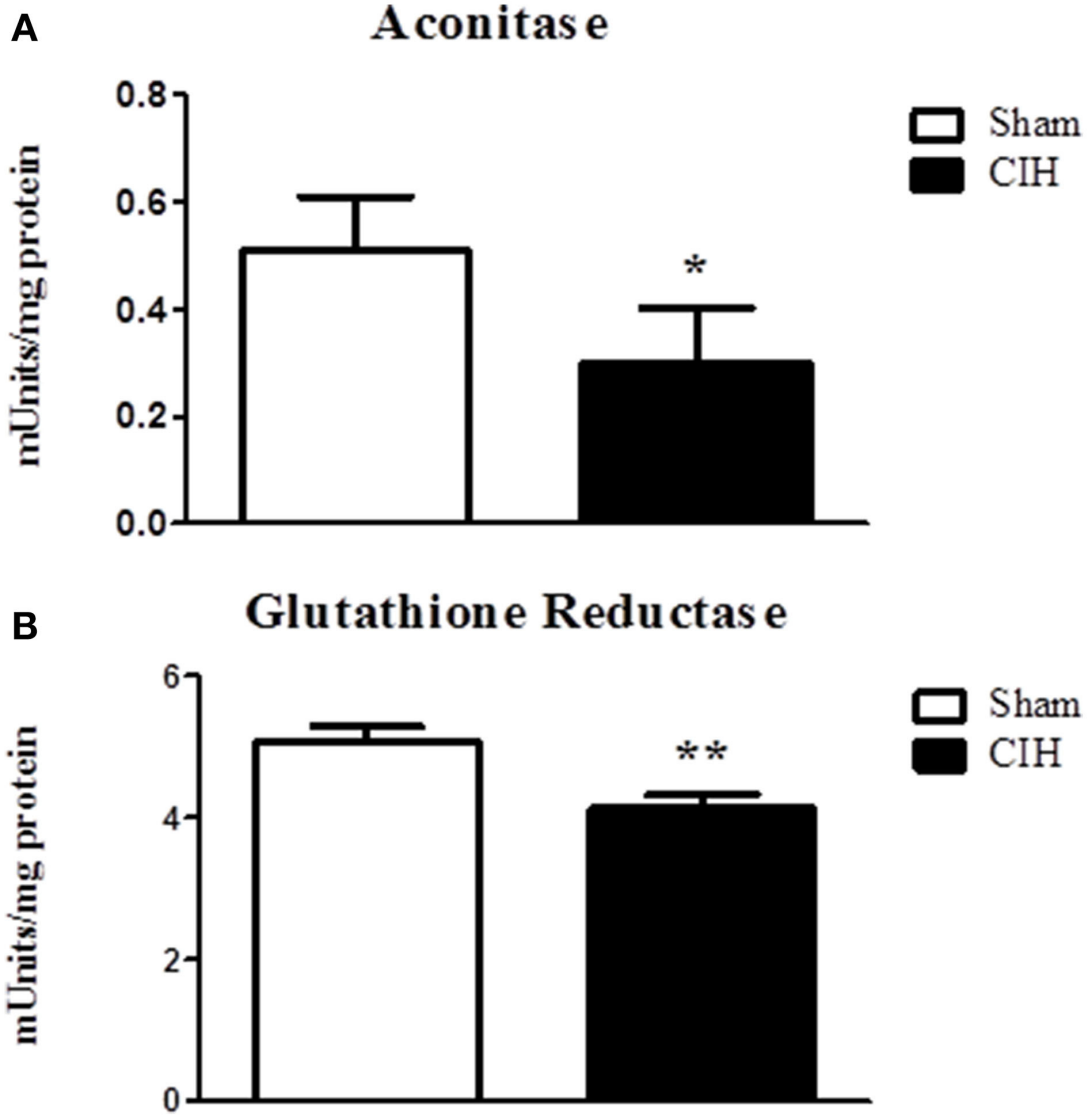
**Sternohyoid muscle aconitase and glutathione reductase enzyme activities in sham and chronic intermittent hypoxia (CIH)-exposed rats**. Aconitase **(A)** and glutathione reductase **(B)** activities were significantly decreased in CIH-exposed sternohyoid muscles compared to sham controls: ^*^P = 0329 and ^**^P = 0.0046; Student unpaired *t*-tests. Values are mean ± SEM; *n* = 8 per group.

We postulated that CIH would decrease aconitase and glutathione reductase activities indicative of oxidative stress. CIH exposure significantly decreased aconitase (Figure 3A) and glutathione reductase (Figure 3B) activities.

### Effect of CIH on sternohyoid muscle HIF-1α content and oxidative and glycolytic enzyme activities

We postulated that CIH would increase HIF-1α content and cause a shift from oxidative-to-glycolytic metabolism. CIH exposure did not affect sternohyoid HIF-1α content (not shown). Similarly, CIH exposure did not affect citrate synthase (0.23 ± 0.01 vs. 0.22 ± 0.01, mean±SEM, units/mg protein; Student unpaired *t*-test: *p* = 0.518), aldolase (1.90 ± 0.06 vs. 2.00 ± 0.11 units/mg protein; *p* = 0.425), lactate dehydrogenase (0.45 ± 0.08 vs. 0.42 ± 0.08 units/mg protein; *p* = 0.805) or glyceraldehyde-3-phosphate dehydrogenase (0.56 ± 0.12 vs. 0.53 ± 0.09 units/mg protein; *p* = 0.845) activities.

### Effect of apocynin on sternohyoid muscle contractile properties

**Figure 4:**
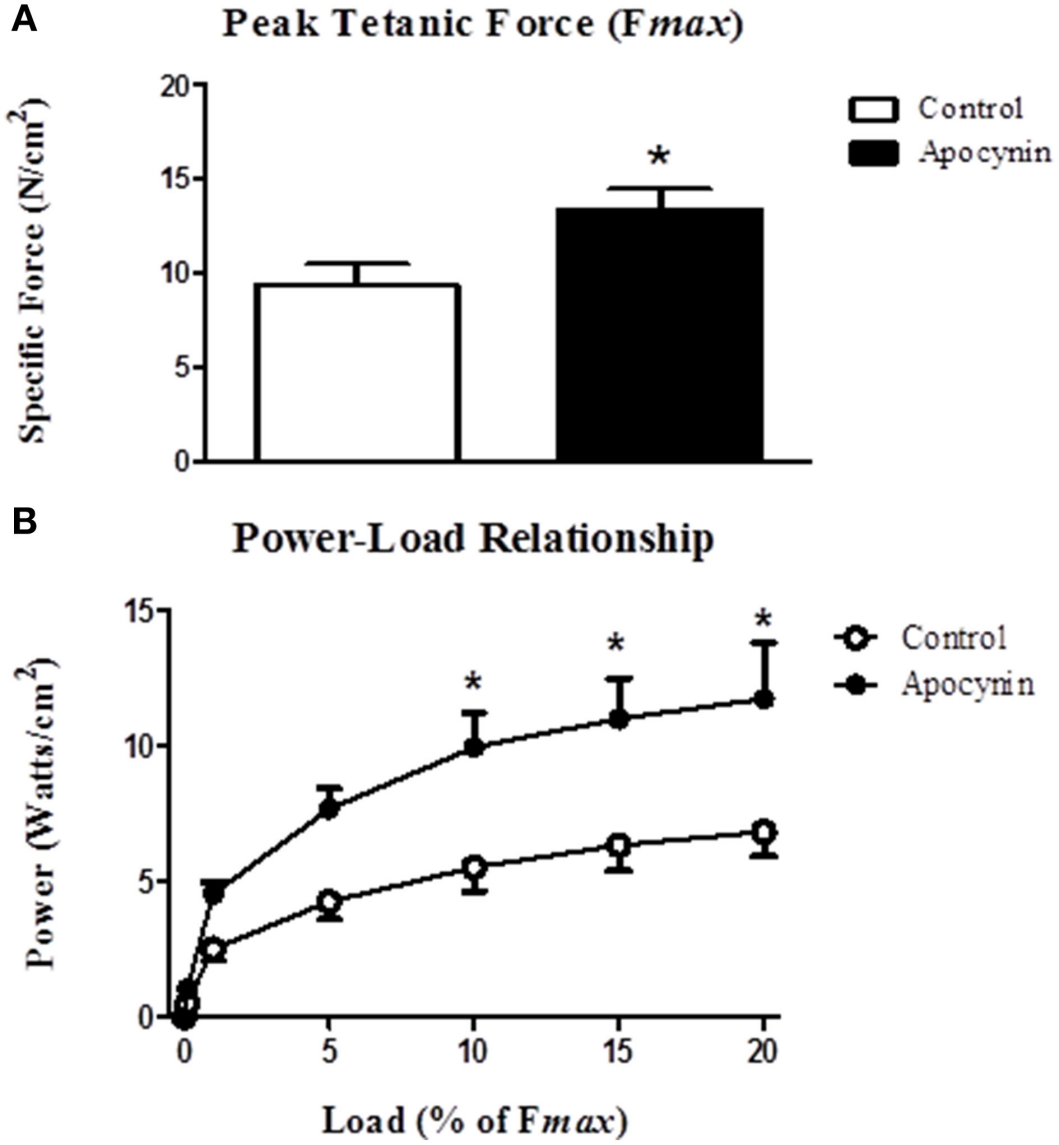
**Sternohyoid muscle isometric and isotonic contractile properties in the absence and presence of apocynin**. **(A)** Sternohyoid peak tetanic force (F*max*) determined *ex vivo* in the absence or presence of 2 mM apocynin (putative NOX inhibitor). F*max* was significantly increased in apocynin-treated muscle preparations (^*^P = 0.03; Student unpaired *t*-test). **(B)** Power-load relationship of sternohyoid muscle determined *ex vivo* in the absence or presence of 2 mM apocynin. Power (force x velocity of shortening) was significantly increased in apocynin-treated preparations (*P* = 0.023 (drug); Two Way repeated measures ANOVA; ^*^P < 0.01 Bonferroni *post-hoc* test). Values are mean ± SEM; *n* = 5 per group.

We postulated that the putative NOX inhibitor—apocynin—would increase force- and power-generating capacity of the sternohyoid muscle. Data for sternohyoid muscle contractile properties are shown in Table 1. Contractile kinetics and twitch force determined during isometric contractions were unaffected by apocynin. There was an increase in the maximum velocity of shortening (V*max*) in apocynin-treated preparations during isotonic contractions but this did not achieve statistical significance (*p* = 0.095). Maximum muscle shortening during isometric contractions (S*max*) was unaffected by apocynin. Conversely, apocynin significantly increased sternohyoid muscle peak tetanic force (F*max*; Figure 4A) determined under isometric conditions and increased muscle power (force x velocity of shortening; Figure 4B) over a range of loads (10–20% of F*max*) during concentric contractions.

**Table 1:** **Sternohyoid muscle contractile properties in the absence and presence of 2 mM apocynin**.

	**Control**	**Apocynin**	***P*-value**
CT (ms)	15 ± 1	17 ± 2	0.407^a^
½RT (ms)	12 ± 1	12 ± 2	0.647^a^
Pt (N/cm^2^)	2.5 ± 0.6	2.6 ± 0.2	0.922^b^
V*max* (Lo/s)	6.5 ± 0.8	8.9 ± 0.7	0.095^c^
S*max* (L/Lo)	0.29 ± 0.02	0.34 ± 0.03	0.129^a^

Values are mean ± SEM; n = 5 for both groups. CT, isometric contraction time; ½RT, isometric half-relaxation time; Pt, isometric twitch force; Vmax, maximum shortening velocity under isotonic conditions of zero load normalized to optimum length (Lo); Smax, peak shortening normalized to Lo. Data were compared using

a Student unpaired t-test,

b Student unpaired t-test with Welch's correction or

c Mann Whitney U-test as appropriate following tests for normality and equal variances in the data sets.

## Discussion

The key findings of this study are: (1) CIH significantly increased NOX 2 and p47 phox—but not p22 phox—subunit expression in rat sternohyoid muscle; (2) CIH did not increase the levels of sternohyoid 4-HNE protein adducts; (3) CIH did not affect protein free thiol or carbonyl group content in sternohyoid muscle; (4) CIH decreased aconitase and glutathione reductase activities indicative of modest oxidative stress; (5) CIH did not affect HIF-1α content or the activities of citrate synthase, aldolase, lactate dehydrogenase or glyceraldehyde-3-phosphate dehydrogenase; (6) The putative NOX inhibitor—apocynin—significantly increased sternohyoid force and power.

It is well established in various animal models that CIH impairs respiratory muscle function (Dunleavy et al., 2008; Skelly et al., 2010, 2012a; Shortt et al., 2014). We have previously reported sternohyoid muscle weakness in adult male Wistar rats, an effect that was ameliorated by chronic tempol (a superoxide scavenger) supplementation (Skelly et al., 2012a) implicating ROS in CIH-induced muscle dysfunction. A variety of sources of ROS have been described but the major contributing source in CIH-induced UA muscle dysfunction has yet to be determined. A number of models have identified the NOX complex as a potential source of ROS contributing to redox modulation and/or oxidative injury (Pigeolet et al., 1990; Berry et al., 2000; Ozaki et al., 2000; Javesghani et al., 2002; Hidalgo et al., 2006; Nair et al., 2011; Shortt et al., 2014). Xia et al. (2003) identified a much higher rate of superoxide production per milligram of protein in the sarcoplasmic reticulum compared to the mitochondria in muscle. In view of these studies and our previous work (Skelly et al., 2010, 2012a,b), we sought to investigate NOX as a potential source of ROS in CIH-exposed UA muscle.

The present study shows that CIH exposure increased key NOX subunits in sternohyoid muscle. We observed a near 3-fold increase in the catalytic subunit—NOX 2—and a near 2-fold increase in the organizer subunit—p47 phox. There is a body of research that implicates hypoxia as a driving force underpinning enhanced NOX expression and activity in various tissues. Acute repetitive hypoxia was shown to increase NOX activity ~12-fold in the carotid body (Peng et al., 2009). This increase in activity was associated with an increase in NOX mRNA and protein expression. CIH was also shown to increase NOX 2 protein and mRNA expression in PC12 pheochromocytoma cells as well as mouse embryonic fibroblasts mediated through hypoxia inducible factor 1 (HIF-1) (Yuan et al., 2011). Similarly, Souvannakitti et al. (2010) showed that CIH increases NOX 2 activity and mRNA expression in adrenal medullae of neonatal rats. Recent work by Zhou et al. (2012) examined the effects of different CIH paradigms, reporting time- and intensity-dependent increases in rat myocardial p22 phox mRNA expression. Extending this line of enquiry, ours is the first study to our knowledge to report an increase in NOX 2 and p47 phox protein subunits in CIH-exposed UA respiratory muscle and this appears to be independent of HIF-1α activation. We acknowledge, however, that the exact site(s) of NOX subunit upregulation is not known and it could extend to the muscle vasculature; this is a limitation of the experimental approach taken in the present study.

Animal models of CIH most often report evidence of increased oxidative stress in various tissues, consistent with the observation that human OSAS is an oxidative stress disorder (Lavie, 2003). Despite reporting a significant increase in NOX subunit expression in CIH-exposed sternohyoid muscle, we did not detect overt oxidative stress in the tissue, assessed by measurement of protein free thiol and carbonyl group content. This was further corroborated by assessment of the levels of 4-HNE (a highly reactive lipid peroxidation product and initiator of oxidative stress) which was unchanged in the CIH-exposed airway dilator muscle. On the face of it, the apparent lack of oxidative stress following CIH is surprising given our previous findings that antioxidant supplementation ameliorates or prevents CIH-induced respiratory muscle dysfunction (Dunleavy et al., 2008; Skelly et al., 2012a; Shortt et al., 2014). Of note however, CIH-induced sternohyoid muscle dysfunction is reversible by acute antioxidant treatment following CIH exposure (Skelly et al., 2012a). The latter observation strongly suggests that CIH exposure does not cause irreversible oxidative modification (damage) to muscle, consistent with the lack of evidence of increased protein carbonylation in CIH-exposed muscle reported in the present study. Rather, CIH-induced sternohyoid muscle weakness (Skelly et al., 2012a) appears to be a dynamic ROS-dependent process that is entirely reversible, which is important in the context of potential antioxidant pharmacotherapy for human OSAS. Of note, CIH was associated with significant decreases in aconitase and glutathione reductase activities highlighting the development of a modest oxidative stress in CIH-exposed muscle.

In view of the collective data described above, we speculate that CIH-induced sternohyoid dysfunction (Skelly et al., 2012a) is mediated by altered redox signaling, perhaps within microdomains of the muscle, evidently with no widespread cellular stress. A number of groups have reported localization of the NOX subunit complex, or at least some components, to areas near the sarcoplasmic reticulum and transverse tubules of muscle (Xia et al., 2003; Hidalgo et al., 2006; Sun et al., 2011). Oxidation of redox-sensitive cysteine residues in these microdomains affects calcium release through ryanodine receptor channels (Hidalgo et al., 2006; Sun et al., 2011); Ca^2+^ release is promoted during low level ROS turnover but is inhibited during high level ROS production (Geiszt et al., 1997; Kawakami and Okabe, 1998; Reid, 2001). Thus, it is plausible to suggest that CIH-induced NOX-dependent elevated ROS might impair muscle force-generating capacity without concomitant widespread oxidative stress/injury. CIH-induced oxidative damage has been observed in skeletal muscle (Dutta et al., 2008) and other tissues (Veasey et al., 2004; Raghuraman et al., 2009; Khan et al., 2011). It appears that the detrimental effects of CIH manifest in a “dose”-dependent manner and the effects of oxidative stress may be organ-specific (Shan et al., 2007; Jun et al., 2008). It is worth noting that the experimental paradigm of CIH used in our studies is relatively modest and based on the observations of Raghuraman et al. (2009), that short bouts of IH (15 s hypoxia; 5 min normoxia) produce higher levels of ROS compared to 90 s hypoxic/normoxic cycles, our paradigm might not be expected to produce severe oxidative stress *per se*.

To explore the potential for NOX-dependent ROS to modulate sternohyoid muscle performance, we examined the effects of apocynin on sternohyoid muscle contractile properties. Apocynin significantly increased isometric sternohyoid peak force revealing that basal NOX-derived ROS exert a powerful inhibitory influence on sternohyoid contractile performance. This finding is consistent with a previous study by our group highlighting that superoxide scavengers are powerful inotropic agents (Skelly et al., 2010, 2012a), extending this work to suggest that NOX is an important source of basal ROS production in rat sternohyoid muscle. Of note, apocynin did not affect isometric contractile kinetics although an increase in the maximum velocity of shortening under isotonic conditions was observed. This failed to achieve statistical significance but the increase likely contributed to the increase in power-generating capacity observed in apocynin-treated preparations. The greater power-generating capacity of the sternohyoid over physiological loads (predominantly due to increased force-generating capacity) translates *in vivo* to a greater capacity to preserve and maintain UA caliber, given that the sternohyoid is a recognized UA dilator muscle. Our data highlight that ROS are powerful inhibitors of sternohyoid force and power, and by extension we posit that increased ROS associated with CIH (or other stimuli including enhanced muscle activity) impairs sternohyoid muscle function. The significance of our findings, in concert with our previous study (Skelly et al., 2012a), is that CIH exposure—a dominant feature of human sleep-disordered breathing—is detrimental to the control of UA caliber, potentially increasing the risk of obstructive airway events. In this regard, CIH exposure could establish an inescapable cycle serving to exacerbate and perpetuate respiratory morbidity in human OSAS.

It is plausible to suggest that NOX-derived ROS could affect contractile function through actions at one or more sites critical in the excitation-contraction coupling mechanism (Geiszt et al., 1997; Kawakami and Okabe, 1998; Reid, 2001; Hidalgo et al., 2006; van der Poel et al., 2007; Lamb and Westerblad, 2011; Sun et al., 2011). It is likely that ROS downstream of superoxide, such as hydrogen peroxide, affect sternohyoid muscle performance (Shortt and O'Halloran, 2014). We speculate that the inotropic effects of apocynin relate to redox modulation of calcium sensitivity of the contractile filaments (Edwards et al., 2007; Murphy et al., 2008). Collectively, the data are consistent with the widely held view that ROS are important signaling molecules in the context of skeletal muscle function. By extension, we postulate that increased NOX-derived ROS production following CIH exposure is likely responsible for sternohyoid muscle weakness (Skelly et al., 2012a), given that ROS are inhibitory to sternohyoid force (this study; Skelly et al., 2010, 2012a,b).

In summary, we have previously shown that CIH exposure causes upper airway dilator muscle weakness, an effect which is blocked by chronic antioxidant supplementation. Here we report that CIH exposure results in increased sternohyoid muscle NOX 2 and p47 phox subunit expression with attendant modest oxidative stress. Apocynin significantly enhanced sternohyoid muscle force- and power-generating capacity. Our results implicate NOX as a potential source of ROS which exert substantial inhibitory effects on sternohyoid muscle performance. We speculate that CIH-induced muscle dysfunction relates to NOX-dependent redox modulation within specific myocellular microdomains relevant to contractile function. The results are further supportive of our previous contention that antioxidant treatment may serve as a useful adjunctive therapy in human OSAS.

## Author contributions

Fiona B. McDonald and Eric Lucking generated the animal model; Robert Williams and Vincent Healy performed electrophoresis and densitometry; Paul Lemaire and Philip Lewis performed enzyme assays; Sean Hogan and Philip Lewis performed muscle physiology experiments; David Sheehan provided advice and expertise on 1D electrophoresis experiments which were performed in his laboratory; all authors contributed to interpretation of the data sets; Ken D. O'Halloran conceived the idea of the study and designed the experiments; Robert Williams drafted the original manuscript; critical revision was provided by David Sheehan, Vincent Healy and Ken D. O'Halloran; all authors approved the final version of the manuscript.

### Conflict of interest statement

The authors declare that the research was conducted in the absence of any commercial or financial relationships that could be construed as a potential conflict of interest.
